# A Simple Microbiome in the European Common Cuttlefish, *Sepia officinalis*

**DOI:** 10.1128/mSystems.00177-19

**Published:** 2019-05-14

**Authors:** Holly L. Lutz, S. Tabita Ramírez-Puebla, Lisa Abbo, Amber Durand, Cathleen Schlundt, Neil R. Gottel, Alexandra K. Sjaarda, Roger T. Hanlon, Jack A. Gilbert, Jessica L. Mark Welch

**Affiliations:** a Department of Pediatrics, University of California—San Diego, La Jolla, California, USA; b Scripps Institution for Oceanography, University of California—San Diego, La Jolla, California, USA; c Integrative Research Center, Field Museum of Natural History, Chicago, Illinois, USA; d Marine Biological Laboratory, Woods Hole, Massachusetts, USA; e Biological Sciences Division, University of Chicago, Chicago, Illinois, USA; University of Wisconsin—Madison

**Keywords:** Cephalopoda, *Piscirickettsiaceae*, *Vibrionaceae*, enrofloxacin, fluorescence assays, microbiome

## Abstract

The European common cuttlefish, Sepia officinalis, is used extensively in biological and biomedical research, yet its microbiome remains poorly characterized. We analyzed the microbiota of the digestive tract, gills, and skin in mariculture-raised *S. officinalis* using a combination of 16S rRNA amplicon sequencing, quantitative PCR (qPCR), and fluorescence spectral imaging. Sequencing revealed a highly simplified microbiota consisting largely of two single bacterial amplicon sequence variants (ASVs) of *Vibrionaceae* and *Piscirickettsiaceae*. The esophagus was dominated by a single ASV of the genus *Vibrio*. Imaging revealed bacteria in the family *Vibrionaceae* distributed in a discrete layer that lines the esophagus. This *Vibrio* was also the primary ASV found in the microbiota of the stomach, cecum, and intestine, but occurred at lower abundance, as determined by qPCR, and was found only scattered in the lumen rather than in a discrete layer via imaging analysis. Treatment of animals with the commonly used antibiotic enrofloxacin led to a nearly 80% reduction of the dominant *Vibrio* ASV in the esophagus but did not significantly alter the relative abundance of bacteria overall between treated versus control animals. Data from the gills were dominated by a single ASV in the family *Piscirickettsiaceae*, which imaging visualized as small clusters of cells. We conclude that bacteria belonging to the *Gammaproteobacteria* are the major symbionts of the cuttlefish *Sepia officinalis* cultured from eggs in captivity and that the esophagus and gills are major colonization sites.

**IMPORTANCE** Microbes can play critical roles in the physiology of their animal hosts, as evidenced in cephalopods by the role of *Vibrio* (*Aliivibrio*) *fischeri* in the light organ of the bobtail squid and the role of *Alpha*- and *Gammaproteobacteria* in the reproductive system and egg defense in a variety of cephalopods. We sampled the cuttlefish microbiome throughout the digestive tract, gills, and skin and found dense colonization of an unexpected site, the esophagus, by a microbe of the genus *Vibrio*, as well as colonization of gills by *Piscirickettsiaceae*. This finding expands the range of organisms and body sites known to be associated with *Vibrio* and is of potential significance for understanding host-symbiont associations, as well as for understanding and maintaining the health of cephalopods in mariculture.

## Abstract (precis)

Microbes can play critical roles in the physiology of their animal hosts, as evidenced in cephalopods by the role of *Vibrio* (*Aliivibrio*) *fischeri* in the light organ of the bobtail squid and the role of *Alpha*- and *Gammaproteobacteria* in the reproductive system and egg defense in a variety of cephalopods. We sampled the cuttlefish microbiome throughout the digestive tract, gills, and skin and found dense colonization of an unexpected site, the esophagus, by a microbe of the genus *Vibrio*, as well as colonization of gills by *Piscirickettsiaceae*. This finding expands the range of organisms and body sites known to be associated with *Vibrio* and is of potential significance for understanding host-symbiont associations, as well as for understanding and maintaining the health of cephalopods in mariculture.

## INTRODUCTION

Symbiotic associations between invertebrates and bacteria are common. Among cephalopods, the most intensely studied association is the colonization of the light organ of the bobtail squid, Euprymna scolopes, by the bioluminescent bacterium *Vibrio* (*Aliivibrio*) *fischeri* in a highly specific symbiosis (1). A more diverse but still characteristic set of bacteria colonize the accessory nidamental gland, from which they are secreted into the egg jelly coat and likely protect the eggs from fungal and bacterial attack (2). The accessory nidamental gland and egg cases of the squid *Doryteuthis* (*Loligo*) *pealeii* and the Chilean octopus (Octopus mimus) have also been reported to contain *Alphaproteobacteria* and *Gammaproteobacteria* (3, 4). These associations indicate that bacteria can play a key role in the physiology of cephalopods.

Sepia officinalis, the European common cuttlefish (hereafter cuttlefish), is used extensively in biological and biomedical research (5 – 7) and is a model organism for the study of rapid adaptive camouflage (8 – 11). Cuttlefish are also widely represented among zoos and aquaria and play an important role in educating the public about cephalopod biology and life history (12). Little is known about the association of bacterial symbionts with cuttlefish and whether such associations may play a role in the health or behavior of these animals. Understanding the importance, or lack thereof, of the cuttlefish microbiome not only will shed light on the basic biology of this model organism but also will have important implications for future husbandry practices and research design.

Using a combination of 16S rRNA amplicon sequencing, fluorescence *in situ* hybridization (FISH), and quantitative PCR (qPCR), we characterized the gastrointestinal tract (GI), gill, skin, and fecal microbiota of the common cuttlefish in wild-bred, captive-raised animals (5) housed at the Marine Biological Laboratory (MBL), Woods Hole, MA. We observed a highly simplified microbiome dominated by *Vibrionaceae* in the gastrointestinal tract and *Piscirickettsiaceae* in the gills. We treated a subset of cuttlefish with the antibiotic enrofloxacin, commonly used among aquarium veterinarians, and found both amplicon sequence variants (ASVs) to remain the dominant bacterial taxa in esophagus and gill microbiota, although *Vibrionaceae*-specific quantitative PCR measures indicated that the overall load of *Vibrio* spp. was significantly reduced with antibiotic treatment. The simplicity of this system makes it a promising model for further exploration of the factors driving host-symbiont associations in marine invertebrates.

## RESULTS

### Two taxa dominate the *S. officinalis* microbiome.

We sampled 27 healthy adult cuttlefish (*Sepia officinalis*) from the mariculture laboratory at the Marine Biological Laboratory (Woods Hole, MA). The study comprised two time periods. The first (20 to 21 June 2017) was a pilot survey in which three individuals were sampled. The second (25 September to 10 October 2017) was an experiment involving 24 individuals, of which 16 were exposed to repeated doses of the antibiotic enrofloxacin and 8 served as untreated controls. 16S rRNA amplicon sequencing of the GI tract, gills, and skin of all 27 animals revealed a highly simplified microbiota dominated by bacterial amplicon sequence variants (ASVs) in the *Vibrionaceae* and *Piscirickettsiaceae* families, regardless of treatment with enrofloxacin.

**FIG 1:**
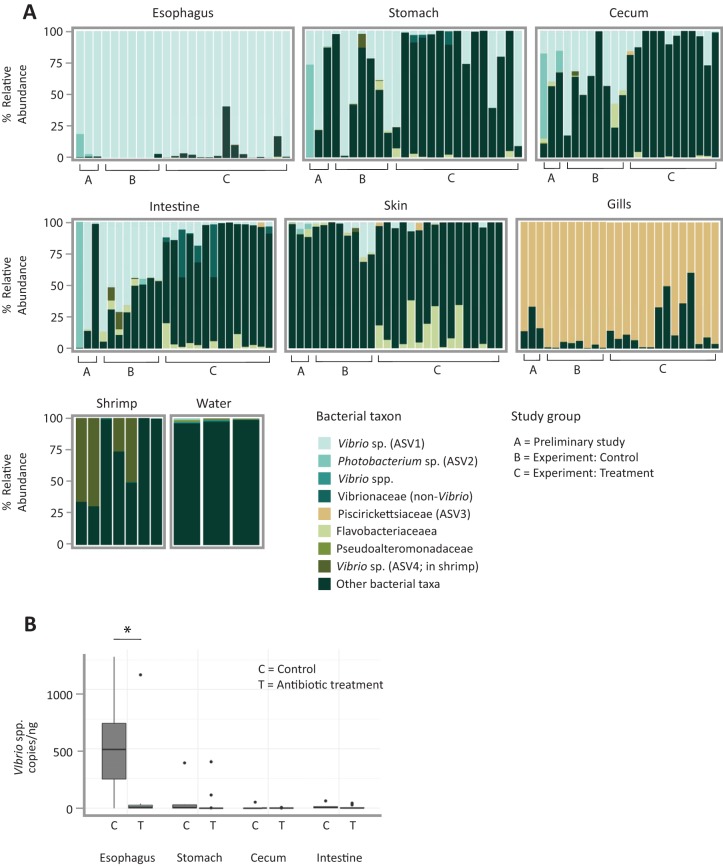
A single *Vibrio* taxon dominates the esophagus, and a single *Piscirickettsiaceae* taxon dominates the gills of the European common cuttlefish in captivity. (A) Relative abundance of top bacterial taxa found among cuttlefish organs. Shrimp for feeding and seawater from holding tanks are also shown. ASVs are labeled according to the finest level of taxonomic resolution provided by the Greengenes database; bacteria not included in the top 8 taxa are pooled as “other.” Bars correspond to individual 16S rRNA sequence libraries from the pilot investigation animals (labeled “A”), experimental animals in the control category (labeled “B”), and experimental animals in the antibiotic treatment category (labeled “C”); only libraries with a read depth of >1,000 are shown. (B) Quantity of *Vibrio* cells per nanogram of DNA measured using *Vibrio*-specific primers (567F and 680R [50]). An asterisk indicates significant difference between organs (*P* < 0.05 by Welch two-sample *t* test).

**FIG 2:**
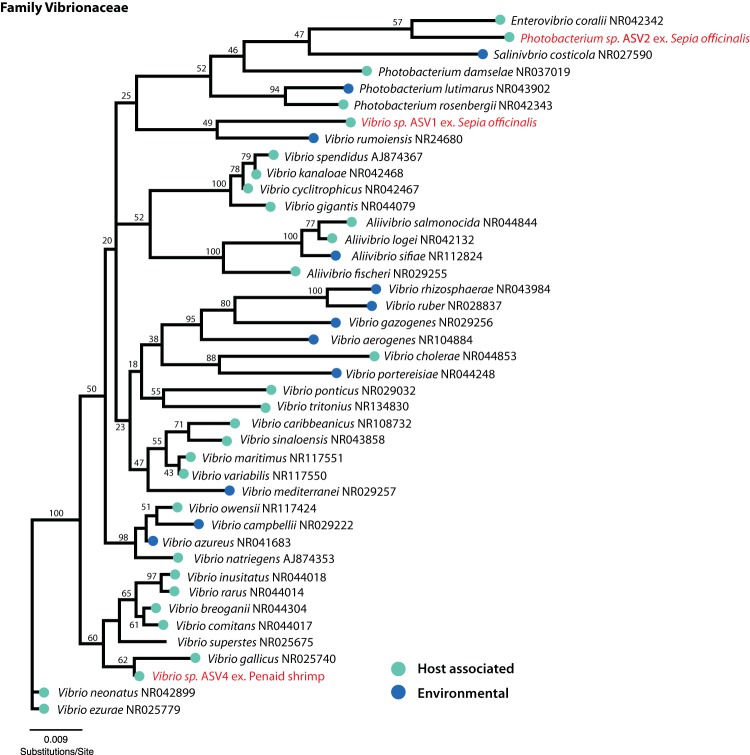
Neighbor-joining tree based on partial 16S rRNA sequences of the family *Vibrionaceae* revealing disparate phylogenetic placement of three major *Vibrionaceae* ASVs identified in this study. GenBank accession numbers for 16S rRNA sequences are listed to the right of each bacterial taxon. ASVs identified in this study are labeled in red text, with “ex.” followed by the host organism with which each was found to be associated. Nodes are labeled with bootstrap support values generated from 100 pseudoreplicates.

In particular, results showed a consistent and highly simplified microbiota in the esophagus (Fig. 1; Table 1). A single ASV in the genus *Vibrio* (referred to as ASV1 in subsequent figures and tables) made up the majority of the 16S rRNA sequence data from the esophagus of the three pilot investigation individuals (mean ± standard deviation [SD] of 92% ± 10%) and of 24 individuals sampled 4 months later (control group, 100% ± 1%; treatment group, 94% ± 10%). Thus, this ASV represents a dominant constituent of the esophagus microbiota stably over two time periods in the study and after exposure to antibiotic treatment with enrofloxacin. Another ASV putatively of the related genus *Photobacterium* (*Vibrionaceae*) (referred to as ASV2 in Table 1) was present in the esophagus community in the pilot investigation animals (7% ± 10%). Combined, the two *Vibrionaceae* ASVs (ASV1 and ASV2) in the three pilot animals constituted >99% of the esophagus community. A phylogenetic analysis of *Vibrionaceae* ASVs identified in this study suggested disparate phylogenetic origins of each ASV, although most nodes differentiating these taxa were not well supported (Fig. 2).

**TABLE 1:** Relative abundance of the three most abundant ASVs found among two sampling periods of the European common cuttlefish

Anatomical site	Relative abundance of^*a*^:								
	ASV1 (*Vibrionaceae*, *Vibrio* sp.)			ASV2 (*Vibrionaceae*, *Photobacterium* sp.)			ASV3 (*Piscirickettsiaceae*, sp. unknown)		
	Pilot	Control	Antibiotic	Pilot	Control	Antibiotic	Pilot	Control	Antibiotic
Esophagus	0.92 ± 0.10	1.00 ± 0.01	0.94 ± 0.10	0.07 ± 0.10	0 ± 0	0 ± 0	0 ± 0	0 ± 0	0 ± 0
Stomach	0.39 ± 0.34	0.43 ± 0.37	0.19 ± 0.31	0.25 + 0.42	0 ± 0	0 ± 0	0 ± 0	0 ± 0	0 ± 0
Cecum	0.24 ± 0.14	0.44 ± 0.23	0.06 ± 0.09	0.28 + 0.35	0 ± 0	0 ± 0	0 ± 0	0 ± 0	0 ± 0
Intestine	0.22 ± 0.42	0.57 ± 0.16	0.06 ± 0.06	0.50 ± 0.57	0.01 ± 0.01	0 ± 0	0 ± 0	0 ± 0	0 ± 0
Gills	0 ± 0	0 ± 0	0 ± 0	0 ± 0	0.01 ± 0.01	0 ± 0	0.79 ± 0.11	0.97 ± 0.03	0.82 ± 0.19
Skin	0.02 ± 0.03	0.08 ± 0.11	0.01 ± 0.02	0.03 ± 0.02	0 ± 0	0 ± 0	0 ± 0	0 ± 0	0.01 ± 0.02

a Averages correspond to relative abundance of individual ASVs from each anatomical site and period. Period 1 consisted of three pilot individuals, and period 2 consisted of eight individuals in the control group and 16 in the antibiotic-treated group.

The major *Vibrio* ASV, ASV1, was also a major constituent of downstream sites in the GI tract, although present at lower abundance measured both as relative abundance in 16S sequencing data and by quantitative PCR (qPCR) (Fig. 1; Table 1). qPCR revealed a high abundance of *Vibrio* cell copies in the esophagus (average of 520.4 ± 410 copies/ng of total DNA, including host DNA) relative to more distal portions of the GI tract that included stomach, cecum, and intestine (combined average, 25.6 ± 77.8 copies/ng [*P* < 0.005, χ^2^ = 16.5, df = 3, by Kruskal-Wallis test]) (Fig. 1B). Comparison of qPCR measures of ASVs in the genus *Vibrio* between the esophagus of treatment and control animals revealed a striking and significant decrease of nearly 80% in the quantity (*P* < 0.02, df = 10.8, by Welch two-sample *t* test). We did not observe a significant difference in *Vibrio* ASV1 quantity between other organs of the digestive tract with antibiotic treatment (Fig. 1B). Relative abundances of *Vibrio* ASV1 in the esophagus and stomach also did not differ significantly between the antibiotic and control groups (Welch two-sample *t* test, *P* > 0.10), but did differ significantly among the cecum (Welch two-sample *t* test, *P* = 0.00235) and intestine (Welch two-sample *t* test, *P* = 1.99e−05) of treatment versus control groups. Analysis of GI tracts (esophagus, stomach, cecum, and intestine samples combined) between treated versus control animals from the second period of study revealed significant differences in weighted UniFrac β-diversity between the two groups by permutational multivariate analysis of variance [PERMANOVA; probability of the *F* statistic, Pr(>*F*), = 0.006, *F* = 5.63, and df = 1], despite the *Vibrio* ASV1 remaining dominant in most organs. These differences in measured relative abundance and β-diversity may result from stochastic variation in low-abundance sequences, as the non-*Vibrio* portion of the 16S rRNA sequence data from the GI tract consisted of an assortment of taxa that varied between individuals or between the two time points of the study and thus suggested transient organisms rather than stable microbial colonization.

**FIG 3:**
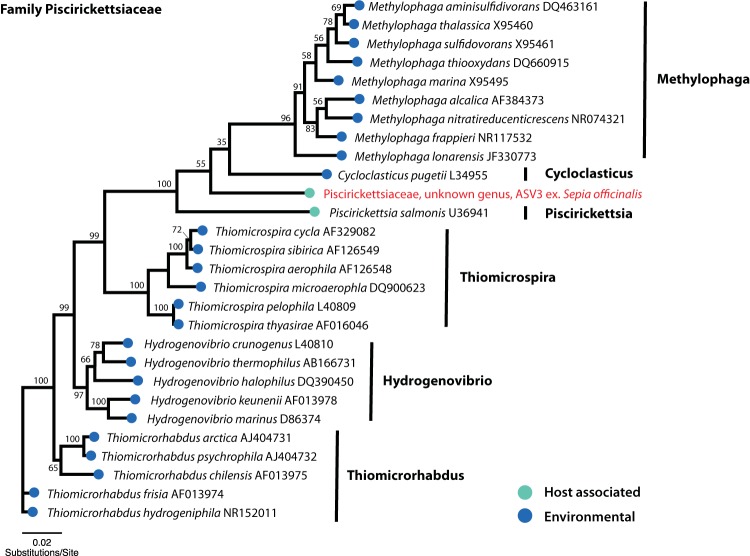
Neighbor-joining tree based on partial 16S rRNA sequences of the family *Piscirickettsiaceae* revealing a sister relationship between a novel *Piscirickettsiaceae* ASV identified in this study and the known fish-associated pathogen Piscirickettsia salmonis. GenBank accession numbers for 16S rRNA sequences are listed to the right of each bacterial taxon, and the ASV identified in this study is labeled in red text with “ex.” followed by the host organism with which it was found to be associated. Nodes are labeled with bootstrap support values generated from 100 pseudoreplicates.

Samples from gills were dominated by a single highly abundant ASV in the family *Piscirickettsiaceae* (referred to as ASV3 in subsequent figures and tables), which made up an average relative abundance of 96.9% ± 2.5% in the gills. Although we did not quantify the abundance of bacteria in the *Piscirickettsiaceae* (i.e., ASV3), an observed decrease in relative abundance of ASV3 in the gills of some antibiotic-treated individuals (Table 1) suggests that this bacterium may be affected to a lesser extent by antibiotics compared to the major reduction measured by qPCR in the *Vibrionaceae*. In samples from other body sites, ASV3 was detected only sporadically and at low relative abundance (mean, 0.2%; range, 0 to 5.8%) (Fig. 1A). Phylogenetic analysis of ASV3 in the context of other recognized bacterial species in the family *Piscirickettsiaceae* placed it as sister to the known pathogen Piscirickettsia salmonis, within the clade also containing the monotypic genus Cycloclasticus pugetii and the genus *Methylophaga*, neither of which is known to contain host-associated bacteria. Despite its position as sister to the known host-associated *Piscirickettsia* (*P. salmonis*), the *Piscirickettsiaceae* ASV3 identified in this study is relatively divergent from other described taxa in our phylogenetic reconstruction, suggesting undescribed diversity within this bacterial clade (Fig. 3).

An additional *Vibrio* ASV, ASV4, was a major constituent of the microbiota of the shrimp used as food for the cuttlefish and was also detectable in some samples from stomach, cecum, and intestine (Fig. 1A). Skin samples did not exhibit much similarity to GI tract or gills with respect to microbiome composition, with most common ASVs found in other anatomical sites comprising <20% (17.2% ± 2.9%) relative abundance of the microbiome (see Table S1 in the supplemental material).

10.1128/mSystems.00177-19.1 TABLE S1 Identity and relative abundance of the top 10 most abundant ASVs across sample types and experimental versus control animals. Download Table S1, XLSX file, 0.07 MB. Copyright © 2019 Lutz et al. 2019 Lutz et al. This content is distributed under the terms of the Creative Commons Attribution 4.0 International license.

**FIG 4:**
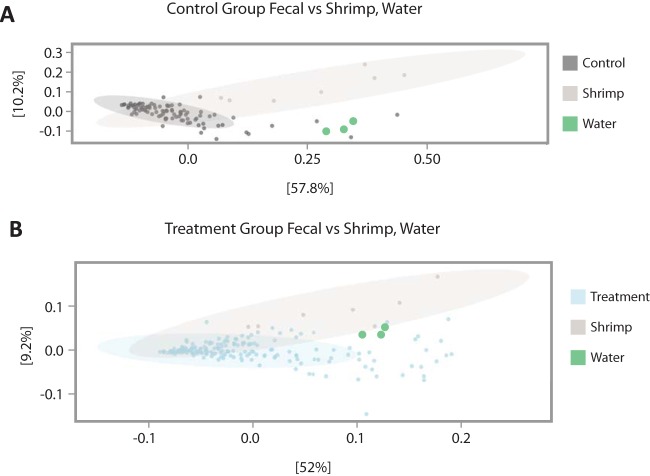
Principal-coordinate analysis (PCoA) of weighted UniFrac β-diversity comparing shrimp, tank water, and (A) fecal samples of treatment cuttlefish and (B) fecal samples of control cuttlefish. Fecal samples from control animals (B) show a more tightly clustered pattern than do fecal samples from animals treated with enrofloxacin (A).

In addition to surveying internal organs and skin, we collected fecal samples from the 24 animals from the second time period of our study. These samples were collected daily for each individual throughout the course of the antibiotic treatment experiment (see Materials and Methods). Comparison of weighted UniFrac dissimilarity of fecal samples from experimental animals revealed significant differences in β-diversity between cuttlefish fecal samples, seawater, and shrimp [PERMANOVA, Pr(>*F*) = 0.001, *F* = 5.26, and df = 2], upon which the animals were fed. These results, paired with the differences we observed in compositional relative abundance between organs, seawater, and shrimp provide additional support for our finding that bacterial communities associated with cuttlefish differ from those found in their seawater environment and food source (Fig. 4).

Imaging shows the spatial structure of the microbiota in cuttlefish esophagus and scattered distribution elsewhere in the gastrointestinal tract.

**FIG 5:**
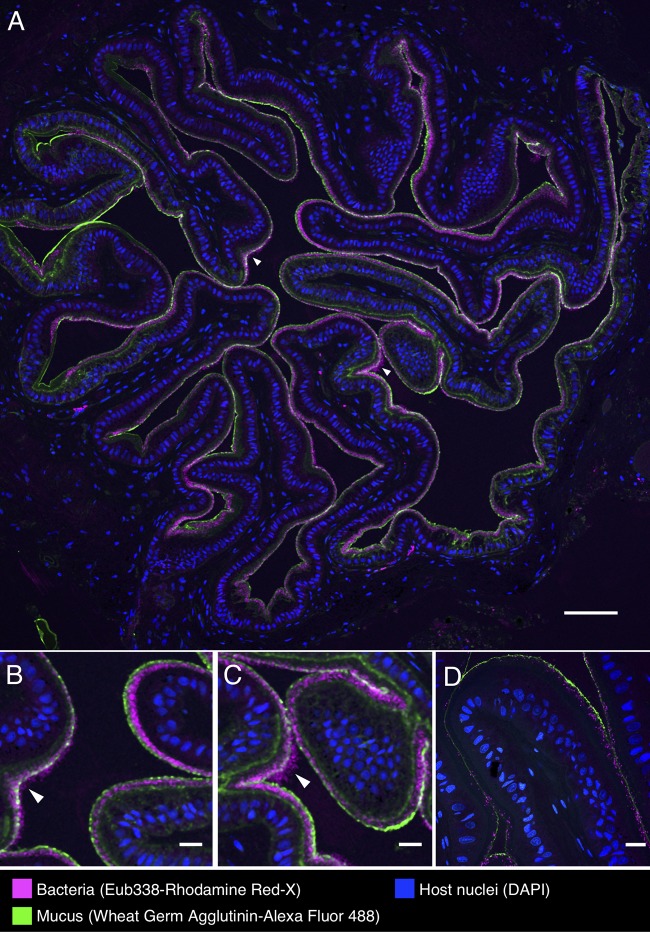
Spatial organization of bacteria in the esophagus of the European common cuttlefish, *S. officinalis.* The images shown are cross-sections of esophagus that were embedded in methacrylate, sectioned, and subjected to fluorescence *in situ* hybridization (FISH) with near-universal bacteria probe (Eub338) and fluorophore-labeled wheat germ agglutinin to visualize mucus. (A) Bacteria (magenta) lining the interior of the esophagus in a control animal in association with the mucus layer (green). Host nuclei (DAPI staining) are shown in blue. Panels B and C are enlarged images of the regions marked with arrowheads in panel A where bacteria extend past the edge of the mucus layer. (D) Esophagus from an antibiotic-treated animal. Scale bars = 100 μm in panel A and 20 μm in panels B, C, and D.

**FIG 6:**
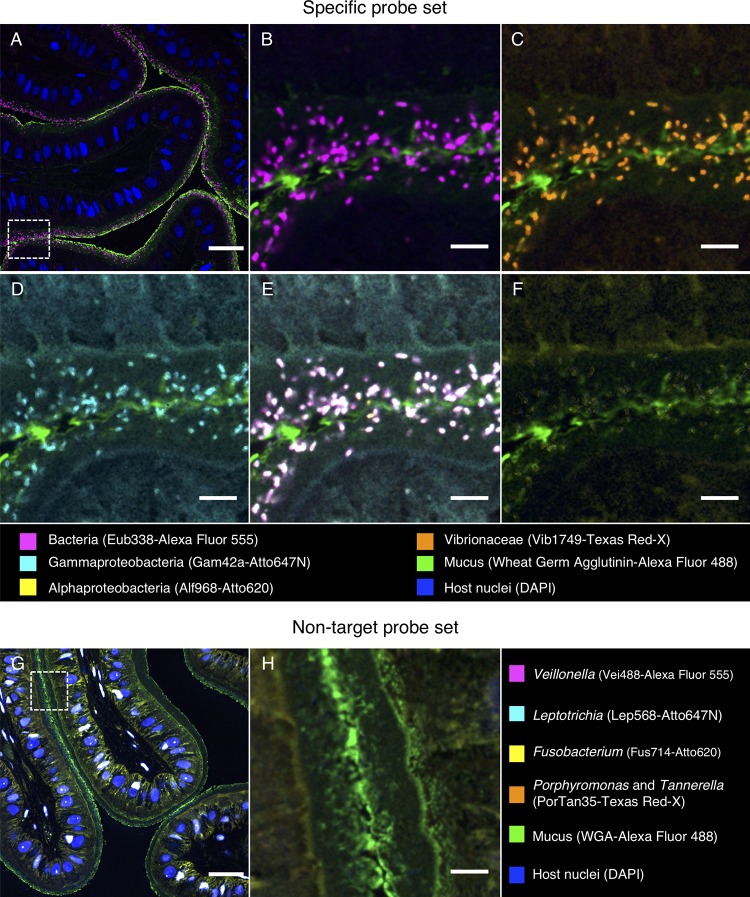
Fluorescence *in situ* hybridization identifies bacteria in the esophagus of *S. officinalis* as *Vibrionaceae*. A methacrylate-embedded section from a control animal was hybridized with a nested probe set containing probes for most bacteria, *Gammaproteobacteria*, *Alphaproteobacteria*, and *Vibrionaceae*. (A) Near-universal probe showing a bacterial distribution similar to that in Fig. 5. (B, C, and D) Enlarged images of the region marked by the dashed square in panel A showing hybridization with near-universal, *Vibrionaceae*, and *Gammaproteobacteria* probes, respectively. (E) Merged image of panels B, C, and D showing an exact match of the signal from those three probes (white). (F) *Alphaproteobacteria* probe showing no hybridization. (G) An independent hybridization with the nontarget probe set as a control. No signal is observed, except for nonspecific binding of probes to host cell nuclei. (H) Enlarged image of the dashed square in panel G. Scale bars = 30 μm in panels A and G and 5 μm in panels B to F and H.

Fluorescence *in situ* hybridization (FISH) revealed a striking organization of bacteria distributed in a layer lining the interior of the convoluted esophagus of cuttlefish (Fig. 5A to C). Hybridization with the near-universal Eub338 probe showed bacteria in high density in a layer ∼20 to 40 μm thick at the border between host tissue and lumen. Staining with fluorophore-conjugated wheat germ agglutinin revealed a mucus layer that covered the epithelium and generally enclosed the bacteria (Fig. 5). To verify the identity of these bacteria, we employed a nested probe set including Eub338, as well as probes for *Alphaproteobacteria* and *Gammaproteobacteria*, and probes we designed specifically for *Vibrionaceae* (Vib1749 and Vib2300 [Table 2]). Bacterial cells imaged in the esophagus showed signal from all probes expected to hybridize with *Vibrionaceae*, suggesting that the bacteria observed in this organ are a near monoculture of this taxon (Fig. 6B to E). A probe targeted to *Alphaproteobacteria* was included in the FISH as a negative control and, as expected, did not hybridize with the cells (Fig. 6F). As an additional control to detect nonspecific binding of probes, we performed an independent FISH with a set of probes labeled with the same fluorophores as the experimental probe set but conjugated to oligonucleotides not expected to hybridize with the cuttlefish microbiota (Table 2). No signal from this nontarget probe set was detected (Fig. 6G and H), supporting the interpretation that the signal observed in the esophagus results from a specific interaction of the *Vibrionaceae*-targeted oligonucleotides with the visualized bacteria.

**TABLE 2:** FISH probes used in this study

Probe	Fluorophore(s)	Target organism	Sequence (5′→3′)	Target position	Reference
Exptl probe set					
Eub338-I	Alexa 555 or Rhodamine Red-X	Most bacteria	GCTGCCTCCCGTAGGAGT	338–355 (16S)	53
Vib1749	Texas Red-X	*Vibrionaceae* family	AGCCACCTGGTATCTGCGACT	1749–1769 (23S)	Unpublished data^*a*^
Vib2300	Texas Red-X	*Vibrionaceae* family	TAACCTCACGATGTCCAACCGTG	2299–2321 (23S)	Unpublished data^*a*^
Alf968	Atto 620 or Dy490	*Alphaproteobacteria*	GGTAAGGTTCTGCGCGTT	968–985 (16S)	54
Gam42a	Atto 647N or Cy5	*Gammaproteobacteria*	GCCTTCCCACATCGTTT	1027–1043 (23S)	55
Nontarget control probes					
Vei488	Alexa 555	*Veillonella*	CCGTGGCTTTCTATTCCG	488–505 (16S)	56
PorTan34	Texas Red-X	*Porphyromonas* and *Tannerella*	GTTAAGCCTATCGCTAGC	34–51 (16S)	Unpublished data^*b*^
Fus714	Atto 620	*Fusobacterium*	GGCTTCCCCATCGGCATT	714–731 (16S)	57
Lep568	Atto 647N	*Leptotrichia*	GCCTAGATGCCCTTTATG	568–585 (16S)	57
Nontarget probe					
Hhaem1007	Rhodamine Red-X	Haemophilus haemolyticus	AGGCACTCCCATATCTCTACAG	1007–1028 (16S)	Unpublished data^*b*^

a C. Schlundt, J. L. Mark Welch, E. R. Zettler, and L. A. Amaral-Zettler.

b J. L. Mark Welch and G. G. Borisy.

**FIG 7:**
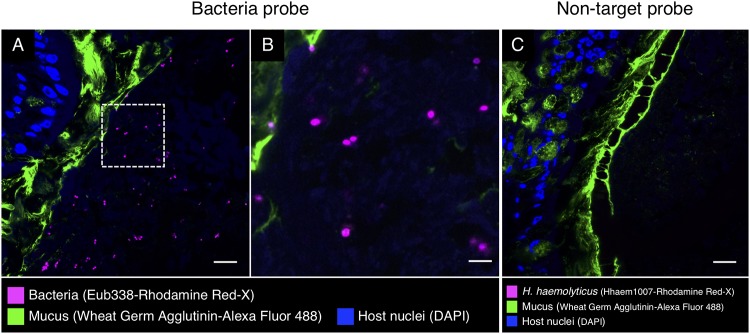
Fluorescence *in situ* hybridization in intestine of *S. officinalis.* Shown is a methacrylate-embedded section of a pilot investigation animal hybridized with the near-universal probe Eub338 and stained with fluorophore-labeled wheat germ agglutinin to visualize mucus. (A) Bacteria (magenta) are sparsely distributed through the lumen. (B) Enlarged image of the dashed square in panel A. (C) An independent FISH control with a nontarget probe (Hhaem1007). No signal was detected. Scale bars = 20 μm in panels A and C and 5 μm in panel B.

**FIG 8:**
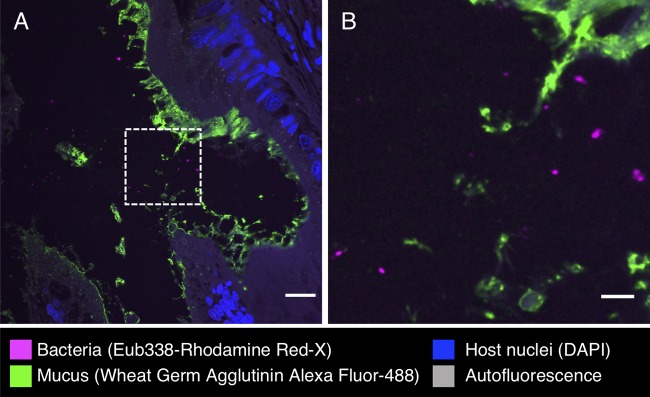
Fluorescence *in situ* hybridization in cecum of *S. officinalis*. (A) Bacteria (magenta) are observed in low abundance in the lumen of cecum of an antibiotic-treated animal. (B) Enlarged image of dashed square in panel A. Scale bars = 20 μm in panel A and 5 μm in panel B.

In other parts of the digestive tract, we observed a sparser distribution of bacteria without obvious spatial organization. Bacteria in the intestine were present not in a layer but scattered throughout the lumen and mixed with the luminal contents (Fig. 7). Similarly, in the cecum, we observed bacteria in low abundance in the lumen (Fig. 8). FISH was also applied to the stomach, posterior salivary gland (poison gland), and duct of the salivary gland, but no bacteria were detected (not shown).

**FIG 9:**
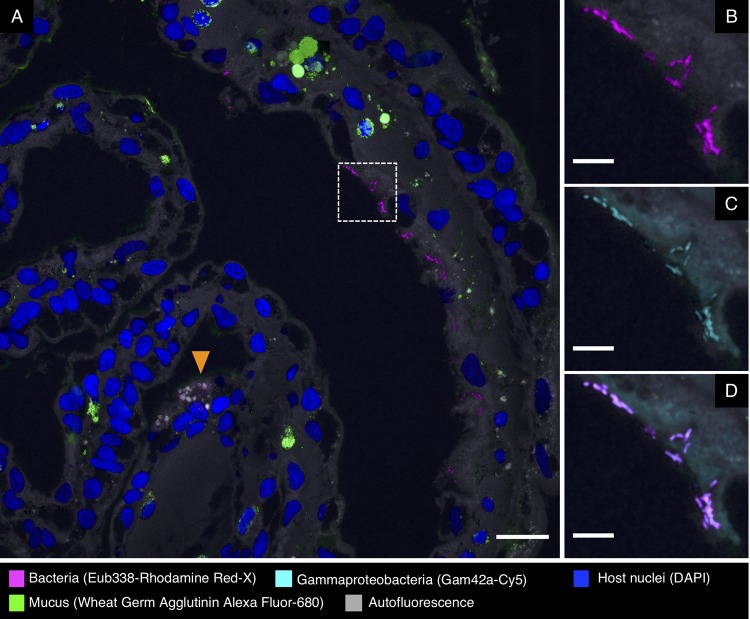
Fluorescence *in situ* hybridization in gills of *S. officinalis*. Bacteria are observed in small clusters at or near the surface of the gill. (A) Overview image of a cross-section from an antibiotic-treated animal. Bacteria are visible as magenta dots in the right half of the image. Many gill sections also contain spherical objects inside host tissue (orange arrowhead) that are autofluorescent and bind to all probes, including nontarget probes from control hybridizations, and we interpret them as nonbacterial inorganic objects. (B and C) Enlarged images of the dashed square in panel A showing bacteria hybridizing with near-universal and *Gammaproteobacteria* probes, respectively. (D) Merged image of panels B and C showing overlap of the signal from those two probes. Scale bars = 20 μm in panel A and 5 μm in panels B to D.

Fluorescence *in situ* hybridization to cross-sections of the gills revealed clusters of bacteria at or near the surface of the tissue (Fig. 9). These bacteria hybridized with the Eub338 near-universal probe and a probe for *Gammaproteobacteria* (Fig. 9B and C; Table 2) but not *Alphaproteobacteria* (not shown), consistent with the identification of the clusters of gill bacteria as members of the gammaproteobacterial family *Piscirickettsiaceae*.

## DISCUSSION

We sampled the cuttlefish microbiome of the digestive tract, gills, and skin and found dense colonization of an unexpected site, the esophagus, by a bacterium of the genus *Vibrio*. Both imaging and 16S rRNA gene sequencing showed a near monoculture of *Vibrionaceae* in the esophagus, with imaging showing dense colonization of the interior lining of the esophagus with a single morphotype that hybridized to probes targeting *Vibrionaceae*. In the remainder of the GI tract, both imaging and 16S rRNA sequencing indicated a less consistent microbiota. Sequencing also showed lower relative abundance of the dominant *Vibrio* ASV, and qPCR confirmed a significantly lower total abundance of *Vibrio* cell copies in the distal GI tract. Imaging revealed sparse and sporadic colonization in the intestine and cecum, with scattered cells in the lumen and no clear colonization of the epithelium. In light of these results, we conclude that the GI tract of laboratory-cultured *Sepia officinalis* has a highly simplified microbiome dominated by the genus *Vibrio*.

Diverse associations with *Vibrio* and the *Vibrionaceae* are known from cephalopods. Among the most extensively investigated is the mutualistic association of the bioluminescent *Vibrio* (*Aliivibrio*) *fischeri* with the light organ of the bobtail squid, *Euprymna scolopes* (1, 13). Other well-known symbioses include the colonization of the cephalopod accessory nidamental gland with *Alpha*- and *Gammaproteobacteria*, which enables the host to secrete a layer of bacteria into the protective coating of the egg capsule (3, 14 – 18). Thus, colonization by *Gammaproteobacteria* and specifically by *Vibrionaceae* is common in cephalopods, yet colonization of the GI tract, and particularly the esophagus, was unexpected.

Bacteria from the genus *Vibrio* and the related *Vibrionaceae* genus *Photobacterium* are frequent colonizers of the GI tracts of marine fishes (19, 20) and are prominent in the gastrointestinal microbiota of Octopus vulgaris paralarvae (21). *Vibrionaceae* have been reported to produce chitinases, proteases, amylase, and lipase (20), suggesting the possibility that colonization of the digestive tract by the *Vibrionaceae* serves to aid in host digestion (20). If the *Vibrio* and *Photobacterium* ASVs serve this function, their localization in high density in the esophagus, near the beginning of the digestive tract, may serve to seed the distal gut; colonization of the lining of the esophagus may provide a reservoir that permits the microbes to avoid washout from the gut by continually repopulating the lumen of downstream gut chambers.

An alternative explanation is that the colonization of the esophagus, and the rest of the gut, is pathogenic or opportunistic. Various *Vibrio* species are known pathogens of cephalopods, causing skin lesions and sometimes mortality in squids and octopuses (22 – 25). The genus *Vibrio* includes representative species that are pathogenic to corals (*V. coralliilyticus*), fish (*V. salmonicida*), diverse marine organisms (V. harveyi), and humans (V. alginolyticus, V. cholerae, V. parahaemolyticus, and V. vulnificus) (26, 27). Likewise, the genus *Photobacterium* contains pathogenic as well as commensal representatives (28). A previous study of the microbiota of *Octopus vulgaris* paralarvae found that recently hatched paralarvae had a high-diversity microbiome that changed, in captivity, to a lower-diversity microbiome with abundant *Vibrionaceae* (21). Whether the *Vibrionaceae* are an integral part of cuttlefish physiology in nature or whether they represent opportunistic colonists of these laboratory-reared organisms is a question for future research.

Our sequence data from gills were dominated by a single ASV classified as *Piscirickettsiaceae* that was in low abundance at other body sites. The *Piscirickettsiaceae* are a family within the *Gammaproteobacteria* (29) that includes the salmon pathogen *P. salmonis*. *Rickettsia*-like organisms have been described from the gills of clams and oysters (30, 31) as well as associated with the copepod Calanus finmarchicus (32). In recent years, *Piscirickettsiaceae* have been identified in high-throughput sequencing data sets from seawater and sediment as a taxon that may be involved in biodegradation of oil and other compounds (33 – 39). Whether taxa in this family colonize the gills of cuttlefish and other organisms as symbionts or as opportunistic pathogens is a subject for future investigation.

Studies of wild *S. officinalis* microbiota will be informative for understanding natural host-symbiont associations under natural conditions, compared to the mariculture-reared animals in the present study. *S. officinalis* cuttlefish in the eastern Atlantic and Mediterranean are known to prey on small mysids (crustaceans) in their first few weeks posthatching; then as juveniles and adults they prey mainly on marine fishes and crabs. The sparseness and simplicity of the gut microbiota observed in our study may have been in part a result of the monodiet of grass shrimp (*Palaemonetes*) we employed. It remains to be seen whether differences in diet and natural variation in environmental conditions influence the association of microbial symbionts with *S. officinalis* in the wild.

Because cuttlefish behavior is well studied and there exist standardized methods for documenting multiple behaviors (8), we hypothesized that these animals may provide a unique opportunity to study microbes and the gut-brain axis—the effect of gut microbiota on behavior (40)—in an invertebrate system. Therefore, in parallel with our study of the microbiome of various organ systems, we conducted extensive preliminary experiments to study the effect of antibiotic treatment on the behavior of *S. officinalis*. These results were largely negative—perhaps due to the highly simplified microbiota we observed and its resilience to the antibiotic employed. These results may prove helpful to the cephalopod husbandry community, as they suggest that application of the commonly used antibiotic enrofloxacin is compatible with maintenance of normal behavioral and microbiome characteristics of this species.

## MATERIALS AND METHODS

### Sampling and antibiotic treatment.

Our study included 27 cuttlefish that were cultured in the laboratory from eggs that were obtained in the wild in southern England and shipped to the Marine Biological Laboratory (MBL). Peak hatching occurred on 28 August 2016. The animals were approximately 13 months old at the time of sampling and were maintained in a subadult (pre-sexually mature) state, ranging in wet weight from 34 to 40 g and with mantle lengths ranging from 70 to 74 mm (see reference 5 for detailed rearing methodology). Animals were held in water tables connected to a single semiclosed filtration system using natural seawater that was filtered only for particulate matter. Animals were euthanized via immersion into a 10% dilution of ethanol in seawater and were then dissected under sterile conditions within a biosafety cabinet using autoclaved tools to obtain samples for microbial analyses. The gastrointestinal tract was dissected into four components: esophagus, stomach, cecum, and intestine. Gill tissue and skin from the mantle were sampled as well (∼0.5 g per sample). All tissues were stored in separate sterile cryogenic tubes and flash-frozen in liquid nitrogen.

Following a pilot study of three individual cuttlefish, we included 24 cuttlefish (16 test, 8 control) in an experiment designed to test the effect of the antibiotic treatment on the composition of the cuttlefish microbiome. The experimental design consisted of administering antibiotic to animals in the treatment group (*n* = 16) via injection into the food source (grass shrimp, *Palaemonetes* sp.), which was then fed to the animals. Prior to feeding, shrimp were injected with enrofloxacin (Baytril; 22.7 mg/ml, [Bayer HealthCare LLC, Shawnee Mission, KS]) using a 0.5-ml, U-100 insulin syringe with an attached 28-gauge 1/2-in. needle (Covidien LLC, Mansfield, MA). The antibiotic dosage was 10 mg/kg of body weight, rounded up to the nearest hundredth milliliter. The antibiotic was injected into the coelomic cavity of the shrimp, which were then immediately fed to the cuttlefish once daily for 14 days. We maintained 8 animals as controls, none of which received antibiotic treatment. Experimental animals were held in two separate water tables and control animals in a third, all of which were connected to the same open-filtration system fed by filtered seawater. Within each water table, animals were isolated into individual holding pens via plastic panels. The experimental period lasted for 14 days (from 1 to 15 Oct 2017), during which fecal samples were collected from each individual daily; fecal samples were collected via pipetting free-floating material from the tank of each animal into a sterile pipette.

To assess the extent to which cuttlefish microbial symbionts were shared with their environment and food sources, 1-liter water samples were taken from each of three water tables in which animals were held on the day of euthanasia and filtered using a 0.22-μm-pore Sterivex filter for DNA extraction; grass shrimp used as the food source throughout the duration of the experiment were collected in 1.8-ml sterile cryotubes on the same day that water was sampled and were frozen at −20˚C until prepared for DNA extraction.

### DNA extraction, sequencing, qPCR, and 16S rRNA gene statistical analyses.

DNA extractions were performed on cuttlefish tissue biopsy specimens, water, and whole shrimp using the MoBio PowerSoil 96-well soil DNA isolation kit (catalog no. 12955-4; MoBio, Carlsbad, CA). We used the standard 515f and 806r primers (41 – 43) to amplify the V4 region of the 16S rRNA gene, using mitochondrial blockers to reduce amplification of host mitochondrial DNA. Sequencing was performed using paired-end 150-base reads on an Illumina HiSeq sequencing platform. Following standard demultiplexing and quality filtering using the Quantitative Insights Into Microbial Ecology pipeline (QIIME2) (44) and vsearch8.1 (45), ASVs were identified using the Deblur method (46), and taxonomy was assigned using the Greengenes database (May 2013 release [http://greengenes.lbl.gov]). Libraries containing fewer than 1,000 reads were removed from further analyses.

Statistical analyses and figure production were carried out with the aid of several R packages, including vegan (47), dplyr (48), ggplot2 (49), and Adobe Illustrator CC 2019. We performed qPCR analyses targeting the genus *Vibrio* to quantify the abundance of bacterial cells throughout the GI tract. Conditions for qPCR were borrowed from Thompson et al. (50), using the primers 567F and 680R and performing two replicates of qPCR analysis that were combined to produce the final results reported in this study.

Phylogenetic analyses were conducted using the program Geneious v7.1.9 (http://www.geneious.com) to assess the phylogenetic placement of major ASVs identified in this study (*Vibrionaceae,* ASV1, ASV2, and ASV4; *Piscirickettsiaceae*, ASV3). Bacterial taxa from the families *Vibrionaceae* and *Piscirickettsiaceae* were selected for inclusion in phylogenetic reconstructions based on their approved standing in the nomenclature (51) and with efforts to include a broad range of representative species (see Table S2 in the supplemental material). Complete or nearly complete 16S rRNA sequences for species from each bacterial family were obtained from NCBI and combined with the Illumina-generated 150-bp partial 16S rRNA sequences from major ASVs found in this study to produce two alignments (one for *Vibrionaceae* and one for *Piscirickettsiaceae*), which we generated using the MUSCLE plugin in Geneious v1.7.9. Each alignment was checked visually for quality, and an unrooted neighbor-joining tree for each bacterial family was generated using the Tamura-Nei model of genetic distance with 100 bootstrap pseudoreplicates. Bacterial taxa and the NCBI accession numbers for sequences included in phylogenetic reconstructions are listed in Table S2.

10.1128/mSystems.00177-19.2 TABLE S2 Bacterial taxa with approved nomenclature from the families *Vibrionaceae* and *Piscirickettsiaceae* included in phylogenetic analyses in this study. NCBI GenBank accession numbers are listed in the first column, and references corresponding to accession numbers are listed in the last column. Unclassified bacterial taxa identified in this study are labeled in red. In some cases, data were generated from type strains maintained by the type culture collections (e.g., American Type Culture Collection), in which the culture collection acronym (e.g., “ATCC”) is listed rather than host or location information. Download Table S2, XLSX file, 0.05 MB. Copyright © 2019 Lutz et al. 2019 Lutz et al. This content is distributed under the terms of the Creative Commons Attribution 4.0 International license.

### Sample collection, fixation, and sectioning for imaging.

Samples from esophagus, stomach, intestine, and cecum of 9 cuttlefish (1 from the pilot study and 8 from the second period experiment) were dissected and divided in order to include the same individuals in both microscopy and sequencing analyses. Immediately after being divided, samples for imaging were fixed with 2% paraformaldehyde in 10 mM Tris (pH 7.5) for 12 h at 4°C, washed in phosphate-buffered saline (PBS), dehydrated through an ethanol series (30, 50, 70, 80, and 100%), and stored at −20°C. For methacrylate embedding, samples were placed in acetone for 1 h, infiltrated with Technovit 8100 glycol methacrylate (EMSdiasum.com) infiltration solution 3 times for 1 h each followed by a final infiltration overnight under vacuum, transferred to Technovit 8100 embedding solution, and solidified for 12 h at 4°C. Blocks were sectioned to a thickness of 5 μm and applied to Ultrastick slides (Thermo Scientific). Sections were stored at room temperature until FISH was performed.

### FISH.

Hybridization solution (900 mM NaCl, 20 mM Tris [pH 7.5], 0.01% SDS, 20% [vol/vol] formamide, with each probe at a final concentration of 2 μM) was applied to sections and incubated at 46°C for 2 h in a chamber humidified with 20% (vol/vol) formamide. Slides were washed in wash buffer (215 mM NaCl, 20 mM Tris [pH 7.5], 5 mM EDTA) at 48°C for 15 min. Samples were incubated with wheat germ agglutinin (20 μg ml^−1^) conjugated with Alexa Fluor 488 and DAPI (4′,6-diamidino-2-phenylindole [1 μg ml^−1^]) at room temperature for 30 min after FISH hybridization to label mucus and host nuclei, respectively. Slides were dipped in ice-cold deionized water, air dried, mounted in ProLong Gold antifade reagent (Invitrogen) with a no. 1.5 coverslip, and cured overnight in the dark at room temperature before imaging. Probes used in this study are listed in Table 2.

### Image acquisition and linear unmixing.

Spectral images were acquired using a Carl Zeiss LSM 780 confocal microscope with a Plan-Apochromat 40×, 1.4 NA objective. Images were captured using simultaneous excitation with 405-, 488-, 561-, and 633-nm laser lines. Linear unmixing was performed with the Zeiss ZEN Black software (Carl Zeiss) using reference spectra acquired from cultured cells hybridized with the Eub338 probe labeled with the appropriate fluorophore and imaged as above. Unmixed images were assembled and false-colored using FIJI software (52).

### Data availability.

All 16S rRNA sequences and sample metadata are publicly available via the QIITA platform under project ID 12365 (http://qiita.ucsd.edu/study/description/12365) and in EBI under accession no. PRJEB32322. Code for analyses and figures is available at www.github.com/hollylutz/CuttlefishMP. Illumina-generated 16S rRNA sequences (150 bp) for major ASVs discussed in this study are also listed in Table S1.

## References

[1] McFall-Ngai M 2014 Divining the essence of symbiosis: insights from the squid-vibrio model. PLoS Biol 12:e1001783. doi:10.1371/journal.pbio.1001783. 24504482 PMC3913551

[2] Kerwin AH, Nyholm SV 2017 Symbiotic bacteria associated with a bobtail squid reproductive system are detectable in the environment, and stable in the host and developing eggs. Environ Microbiol 19:1463–1475. doi:10.1111/1462-2920.13665. 28063183

[3] Barbieri E, Paster BJ, Hughes D, Zurek L, Moser DP, Teske A, Sogin ML 2001 Phylogenetic characterization of epibiotic bacteria in the accessory nidamental gland and egg capsules of the squid Loligo pealei (Cephalopoda: Loliginidae). Environ Microbiol 3:151–167. doi:10.1046/j.1462-2920.2001.00172.x. 11321532

[4] Iehata S, Valenzuela F, Riquelme C 2016 Evaluation of relationship between Chilean octopus (Octopus mimus Gould, 1852) egg health condition and the egg bacterial community. Aquac Res 47:649–659. doi:10.1111/are.12525.

[5] Panetta D, Solomon M, Buresch K, Hanlon RT 2017 Small-scale rearing of cuttlefish (Sepia officinalis) for research purposes. Mar Freshw Behav Physiol 50:115–124. doi:10.1080/10236244.2017.1343631.

[6] Messenger JB 2001 Cephalopod chromatophores: neurobiology and natural history. Biol Rev Camb Philos Soc 76:473–528. doi:10.1017/S1464793101005772. 11762491

[7] Darmaillacq AS, Dickel L, Mather J 2014 Cephalopod cognition. Cambridge University Press, Cambridge, United Kingdom.

[8] Hanlon RT, Messenger JB 1988 Adaptive coloration in young cuttlefish (*Sepia officinalis L*): the morphology and development of body patterns and their relation to behaviour. Philos Trans R Soc B 320:437–487. doi:10.1098/rstb.1988.0087.

[9] Buresch KC, Ulmer KM, Akkaynak D, Allen JJ, Mäthger LM, Nakamura M, Hanlon RT 2015 Cuttlefish adjust body pattern intensity with respect to substrate intensity to aid camouflage, but do not camouflage in extremely low light. J Exp Mar Biol Ecol 462:121–126. doi:10.1016/j.jembe.2014.10.017.

[10] Yu C, Li Y, Zhang X, Huang X, Malyarchuk V, Wang S, Shi Y, Gao L, Su Y, Zhang Y, Xu H, Hanlon RT, Huang Y, Rogers JA 2014 Adaptive optoelectronic camouflage systems with designs inspired by cephalopod skins. Proc Natl Acad Sci U S A 111:12998–13003. doi:10.1073/pnas.1410494111. 25136094 PMC4246966

[11] Chiao CC, Chubb C, Hanlon RT 2015 A review of visual perception mechanisms that regulate rapid adaptive camouflage in cuttlefish. J Comp Physiol A Neuroethol Sens Neural Behav Physiol 201:933–945. doi:10.1007/s00359-015-0988-5. 25701389

[12] Tonkins BM, Tyers AM, Cooke GM 2015 Cuttlefish in captivity: an investigation into housing and husbandry for improving welfare. Appl Anim Behav Sci 168:77–83. doi:10.1016/j.applanim.2015.04.004.

[13] Koch EJ, Miyashiro T, McFall-Ngai MJ, Ruby EG 2014 Features governing symbiont persistence in the squid-vibrio association. Mol Ecol 23:1624–1634. doi:10.1111/mec.12474. 24118200 PMC3907463

[14] Bloodgood RA 1977 The squid accessory nidamental gland: ultrastructure and association with bacteria. Tissue Cell 9:197–208. doi:10.1016/0040-8166(77)90016-7. 906013

[15] Pichon D, Domart-Coulon I, Boucher-Rodoni R 2017 Cephalopod bacterial associations: characterization and isolation of the symbiotic complex in the accessory nidamental glands. Boll Malacol 43:96–102.

[16] Collins AJ, LaBarre BA, Won BS, Shah MV, Heng S, Choudhury MH, Haydar SA, Santiago J, Nyholm SV 2012 Diversity and partitioning of bacterial populations within the accessory nidamental gland of the squid Euprymna scolopes. Appl Environ Microbiol 78:4200–4208. doi:10.1128/AEM.07437-11. 22504817 PMC3370523

[17] Gromek SM, Suria AM, Fullmer MS, Garcia JL, Gogarten JP, Nyholm SV, Balunas MJ 2016 Leisingera sp. JC12, a bacterial isolate from Hawaiian bobtail squid eggs, produces indigoidine and differentially inhibits vibrios. Front Microbiol 7:1342. doi:10.3389/fmicb.2016.01342. 27660622 PMC5014874

[18] Lum-Kong A, Hastings TS 1992 The accessory nidamental glands of Loligo forbesi (Cephalopoda: Loliginidae): characterization of symbiotic bacteria and preliminary experiments to investigate factors controlling sexual maturation. J Zool (Lond) 228:395–403. doi:10.1111/j.1469-7998.1992.tb04443.x.

[19] Tarnecki AM, Burgos FA, Ray CL, Arias CR 2017 Fish intestinal microbiome: diversity and symbiosis unravelled by metagenomics. J Appl Microbiol 123:2–17. doi:10.1111/jam.13415. 28176435

[20] Egerton S, Culloty S, Whooley J, Stanton C, Ross RP 2018 The gut microbiota of marine fish. Front Microbiol 9:837. doi:10.3389/fmicb.2018.00873. 29780377 PMC5946678

[21] Roura A, Doyle SR, Nande M, Strugnell JM 2017 You are what you eat: a genomic analysis of the gut microbiome of captive and wild Octopus vulgaris paralarvae and their zooplankton prey. Front Physiol 8:362. doi:10.3389/fphys.2017.00362. 28620315 PMC5450036

[22] Hanlon RT, Forsythe JW 1990 1. Diseases of Mollusca: Cephalopoda. 1.1. Diseases caused by microorganisms, p 23–46. *In* Kinne O (ed), Diseases of marine animals, vol III Biologische Anstalt Helgoland, Hamburg, Germany.

[23] Forsythe JW, Hanlon RT, Lee PG 1990 A formulary for treating cephalopod mollusc diseases. Academic Press, San Diego, CA.

[24] Ford LA, Alexander SK, Cooper KM, Hanlon RT 1986 Bacterial populations of normal and ulcerated mantle tissue of the squid, Lolliguncula brevis. J Invertebr Pathol 48:13–26. doi:10.1016/0022-2011(86)90138-2. 3722856

[25] Hanlon RT, Forsythe JW 1990 1. Diseases of Mollusca: Cephalopoda. 1.3. Structural abnormalities and neoplasia, p 203–204. *In* Kinne O (ed), Diseases of marine animals, vol III Biologische Anstalt Helgoland, Hamburg, Germany.

[26] Takemura AF, Chien DM, Polz MF 2014 Associations and dynamics of Vibrionaceae in the environment, from the genus to the population level. Front Microbiol 5:38. doi:10.3389/fmicb.2014.00038. 24575082 PMC3920100

[27] Pruzzo C, Huq A, Colwell RR, Donelli G 2005 Pathogenic Vibrio species in the marine and estuarine environment, p 217–252. *In* Belkin S, Colwell RR (ed), Oceans and health: pathogens in the marine environment. Springer US, Boston, MA. doi:10.1007/0-387-23709-7_9.

[28] Labella AM, Arahal DR, Castro D, Lemos ML, Borrego JJ 2017 Revisiting the genus Photobacterium: taxonomy, ecology and pathogenesis. Int Microbiol 20:1–10. doi:10.2436/20.1501.01.280. 28581017

[29] Mauel MJ, Giovannoni SJ, Fryer JL 1999 Phylogenetic analysis of Piscirickettsia salmonis by 16S, internal transcribed spacer (ITS) and 23S ribosomal DNA sequencing. Dis Aquat Organ 35:115–123. doi:10.3354/dao035115. 10092974

[30] Azevedo C, Villalba A 1991 Extracellular giant rickettsiae associated with bacteria in the gill of Crassostrea gigas (Mollusca, Bivalvia). J Invertebr Pathol 58:75–81. doi:10.1016/0022-2011(91)90164-L. 1885924

[31] Wen C-M, Kou GH, Chen SN 1994 Rickettsiaceae-like microorganisms in the gill and digestive gland of the hard clam, Meretrix lusoria Röding. J Invertebr Pathol 64:138–142. doi:10.1006/jipa.1994.1082.

[32] Moisander P, Sexton AD, Meaghan CD 2015 Stable associations masked by temporal variability in the marine copepod microbiome. PLoS One 10:e0138967. doi:10.1371/journal.pone.0138967. 26393930 PMC4579122

[33] Lu X, Sun S, Hollibaugh JT, Mou X 2015 Identification of polyamine-responsive bacterioplankton taxa in South Atlantic Bight. Environ Microbiol Rep 7:831–838. doi:10.1111/1758-2229.12311. 26109269

[34] Wang K, Zhang D, Xiong J, Chen X, Zheng J, Hu C, Yang Y, Zhu J 2015 Response of bacterioplankton communities to cadmium exposure in coastal water microcosms with high temporal variability. Appl Environ Microbiol 81:231–240. doi:10.1128/AEM.02562-14. 25326310 PMC4272717

[35] Hamdan LJ, Salerno JL, Reed A, Joye SB, Damour M 2018 The impact of the Deepwater Horizon blowout on historic shipwreck-associated sediment microbiomes in the northern Gulf of Mexico. Sci Rep 8:9057. doi:10.1038/s41598-018-27350-z. 29955123 PMC6023898

[36] Kamalanathan M, Xu C, Schwehr K, Bretherton L, Beaver M, Doyle SM, Genzer J, Hillhouse J, Sylvan JB, Santschi P, Quigg A 2018 Extracellular enzyme activity profile in a chemically enhanced water accommodated fraction of surrogate oil: toward understanding microbial activities after the Deepwater Horizon oil spill. Front Microbiol 9:798. doi:10.3389/fmicb.2018.00798. 29740422 PMC5928240

[37] Lofthus S, Netzer R, Lewin AS, Heggeset TMB, Haugen T, Brakstad OG 2018 Biodegradation of n-alkanes on oil-seawater interfaces at different temperatures and microbial communities associated with the degradation. Biodegradation 29:141–157. doi:10.1007/s10532-018-9819-z. 29397457

[38] Ribicic D, McFarlin KM, Netzer R, Brakstad OG, Winkler A, Throne-Holst M, Størseth TR 2018 Oil type and temperature dependent biodegradation dynamics—combining chemical and microbial community data through multivariate analysis. BMC Microbiol 18:83. doi:10.1186/s12866-018-1221-9. 30086723 PMC6081865

[39] Wang B, Liu H, Tang H, Hu X 2018 Microbial ecological associations in the surface sediments of Bohai Strait. J Ocean Limnol 36:795–804. doi:10.1007/s00343-018-6289-4.

[40] Carabotti M, Scirocco A, Maselli MA, Severi C 2015 The gut-brain axis: interactions between enteric microbiota, central and enteric nervous systems. Ann Gastroenterol 28:203–209. 25830558 PMC4367209

[41] Caporaso JG, Lauber CL, Walters WA, Berg-Lyons D, Lozupone CA, Turnbaugh PJ, Fierer N, Knight R 2011 Global patterns of 16S rRNA diversity at a depth of millions of sequences per sample. Proc Natl Acad Sci U S A 108:4516–4522. doi:10.1073/pnas.1000080107. 20534432 PMC3063599

[42] Caporaso JG, Lauber CL, Walters WA, Berg-Lyons D, Huntley J, Fierer N, Owens SM, Betley J, Fraser L, Bauer M, Gormley N, Gilbert JA, Smith G, Knight R 2012 Ultra-high-throughput microbial community analysis on the Illumina HiSeq and MiSeq platforms. ISME J 6:1621–1624. doi:10.1038/ismej.2012.8. 22402401 PMC3400413

[43] Kozich JJ, Westcott SL, Baxter NT, Highlander SK, Schloss PD 2013 Development of a dual-index sequencing strategy and curation pipeline for analyzing amplicon sequence data on the MiSeq Illumina sequencing platform. Appl Environ Microbiol 79:5112–5120. doi:10.1128/AEM.01043-13. 23793624 PMC3753973

[44] Caporaso JG, Kuczynski J, Stombaugh J, Bittinger K, Bushman FD, Costello EK, Fierer N, Peña AG, Goodrich JK, Gordon JI, Huttley GA, Kelley ST, Knights D, Koenig JE, Ley RE, Lozupone CA, McDonald D, Muegge BD, Pirrung M, Reeder J, Sevinsky JR, Turnbaugh PJ, Walters WA, Widmann J, Yatsunenko T, Zaneveld J, Knight R 2010 QIIME allows analysis of high-throughput community sequencing data. Nat Methods 7:335–336. doi:10.1038/nmeth.f.303. 20383131 PMC3156573

[45] Rognes T, Flouri T, Nichols B, Quince C, Mahé F 2016 VSEARCH: a versatile open source tool for metagenomics. PeerJ 4:e2584. doi:10.7717/peerj.2584. 27781170 PMC5075697

[46] Amir A, McDonald D, Navas-Molina JA, Kopylova E, Morton JT, Zech Xu Z, Kightley EP, Thompson LR, Hyde ER, Gonzolez A, Knight R 2017 Deblur rapidly resolves single-nucleotide community sequence patterns. mSystems 2:e00191-16. doi:10.1128/mSystems.00191-16. 28289731 PMC5340863

[47] Oksanen J, Blanchet FG, Friendly M, Kindt R, Legendre P, McGlinn D, Minchin PR, O'Hara RB, Simpson GL, Solymos P, Henry M, Stevens H, Szoecs EH 2018 vegan: Community Ecology Package, vR package version 2.5-2. https://CRAN.R-project.org/package=vegan.

[48] Wickham H, François R, Henry L, Müller K 2018 dplyr: a grammar of data manipulation, vR package version 0.7.6. https://CRAN.R-project.org/package=dplyr.

[49] Wickham H 2016 ggplot2: elegant graphics for data analysis. Springer-Verlag, New York, NY.

[50] Thompson J, Randa M, Marcelino L, Tomita-Mitchell A, Lim E, Polz M 2004 Diversity and dynamics of a North Atlantic coastal *Vibrio* community. Appl Environ Microbiol 70:4103–4110. doi:10.1128/AEM.70.7.4103-4110.2004. 15240289 PMC444776

[51] Parte AC 2018 LPSN—List of Prokaryotic names with Standing in Nomenclature (bacterio.net), 20 years on. Int J Syst Evol Microbiol 68:1825–1829. doi:10.1099/ijsem.0.002786. 29724269

[52] Schindelin J, Arganda-Carreras I, Frise E, Kaynig V, Longair M, Pietzsch T, Preibisch S, Rueden C, Saalfeld S, Schmid B, Tinevez JY, White DJ, Hartenstein V, Eliceiri K, Tomancak P, Cardona A 2012 Fiji: an open-source platform for biological-image analysis. Nat Methods 9:676–682. doi:10.1038/nmeth.2019. 22743772 PMC3855844

[53] Amann RI, Binder BJ, Olson RJ, Chisholm SW, Devereux R, Stahl DA 1990 Combination of 16S rRNA-targeted oligonucleotide probes with flow cytometry for analyzing mixed microbial populations. Appl Environ Microbiol 56:1919–1925. 2200342 10.1128/aem.56.6.1919-1925.1990 PMC184531

[54] Neef A 1997 Anwendung der in situ-Einzelzell—Identifizierung von Bakterien zur Populations analyse in komplexen mikrobiellen Biozönosen. University of Munich, Munich, Germany.

[55] Manz W, Amann R, Ludwig W, Wagner M, Schleifer KH 1992 Phylogenetic oligodeoxynucleotide probes for the major subclasses of Proteobacteria: problems and solutions. Systematic and Applied Microbiology 15:593–600. doi:10.1016/S0723-2020(11)80121-9.

[56] Chalmers NI, Palmer RJ Jr, Cisar JO, Kolenbrander PE 2008 Characterization of a Streptococcus sp.-Veillonella sp. community micromanipulated from dental plaque. J Bacteriol 190:8145–8154. doi:10.1128/JB.00983-08. 18805978 PMC2593232

[57] Valm AM, Mark Welch JL, Rieken CW, Hasegawa Y, Sogin ML, Oldenbourg R, Dewhirst FE, Borisy GG 2011 Systems-level analysis of microbial community organization through combinatorial labeling and spectral imaging. Proc Natl Acad Sci U S A 108:4152–4157. doi:10.1073/pnas.1101134108. 21325608 PMC3054005

